# Bioimaging and prospects of night pearls‐based persistence phosphors in cancer diagnostics

**DOI:** 10.1002/EXP.20230124

**Published:** 2024-01-23

**Authors:** Ruipu Shang, Feifei Yang, Ge Gao, Yu Luo, Hongpeng You, Lile Dong

**Affiliations:** ^1^ Key Laboratory of Rare Earths Chinese Academy of Sciences Ganjiang Innovation Academy Chinese Academy of Sciences Ganzhou China; ^2^ University of Science and Technology of China Hefei China; ^3^ Division of Physical Science and Engineering (PSE) King Abdullah University of Science and Technology (KAUST) Thuwal Saudi Arabia; ^4^ Shanghai Engineering Technology Research Center for Pharmaceutical Intelligent Equipment Shanghai Frontiers Science Research Center for Druggability of Cardiovascular noncoding RNA Institute for Frontier Medical Technology College of Chemistry and Chemical Engineering Shanghai University of Engineering Science Shanghai China

**Keywords:** bioimaging, inorganic persistence phosphors, luminescence mechanism, nanomedicine, synthetic strategy

## Abstract

Inorganic persistent phosphors feature great potential for cancer diagnosis due to the long luminescence lifetime, low background scattering, and minimal autofluorescence. With the prominent advantages of near‐infrared light, such as deep penetration, high resolution, low autofluorescence, and tissue absorption, persistent phosphors can be used for deep bioimaging. We focus on highlighting inorganic persistent phosphors, emphasizing the synthesis methods and applications in cancer diagnostics. Typical synthetic methods such as the high‐temperature solid state, thermal decomposition, hydrothermal/solvothermal, and template methods are proposed to obtain small‐size phosphors for biological organisms. The luminescence mechanisms of inorganic persistent phosphors with different excitation are discussed and effective matrixes including galliumate, germanium, aluminate, and fluoride are explored. Finally, the current directions where inorganic persistent phosphors can continue to be optimized and how to further overcome the challenges in cancer diagnosis are summarized.

## Graphical abstract

This review is directed at the bioimaging of persistent phosphors. Typical synthetic methods such as the high‐temperature solid state, thermal decomposition, hydrothermal/solvothermal, and template methods are proposed. The luminescence mechanisms of persistent phosphors are discussed and effective matrixes including galliumate, germanium, aluminate, and fluoride is explored. Finally, persistence phosphors have the potential to improve the signal‐to‐noise ratio and sensitivity in bioimaging.

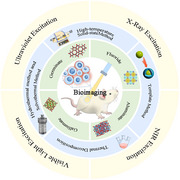

## 1 INTRODUCTION

Persistence phosphors exhibit crucial functions in different areas, such as paintings, decorations, and bioimaging.^[^ ^1^, ^2^, ^3^ ^]^ Persistence phosphors can be divided into inorganic persistence phosphors, and organic persistence phosphors.^[^ ^4^, ^5^ ^]^ Briefly, organic persistence phosphors feature the unfavorable luminescence lifetime, which comes from chemiluminescence. Comparatively, inorganic persistence phosphors possess satisfactory luminescence characteristics such as afterglow intensity and luminous life. Various inorganic persistence phosphors substrate materials have been intensively studied including galliumate,^[^ ^6^ ^]^ germanate,^[^ ^7^ ^]^ aluminate,^[^ ^8^ ^]^ and fluoride.^[^ ^9^ ^]^ Inorganic persistence phosphors consist of a solid matrix with variable storage potential and dopant ions.^[^ ^10^ ^]^ The solid matrix acts as the trapped carrier to delay the electron‐hole complexation process. And the dopant ions are fully utilized to form the luminescence and trap centers.^[^ ^11^ ^]^ The emission wavelength of phosphor is determined by the emitter, and the trap state and distribution affect the intensity and timing of the luminescence. The inorganic persistence phosphors can be endowed with more feasible functions by reasonably designing the structural components, such as available photostability and long afterglow time.

**SCHEME 1:**
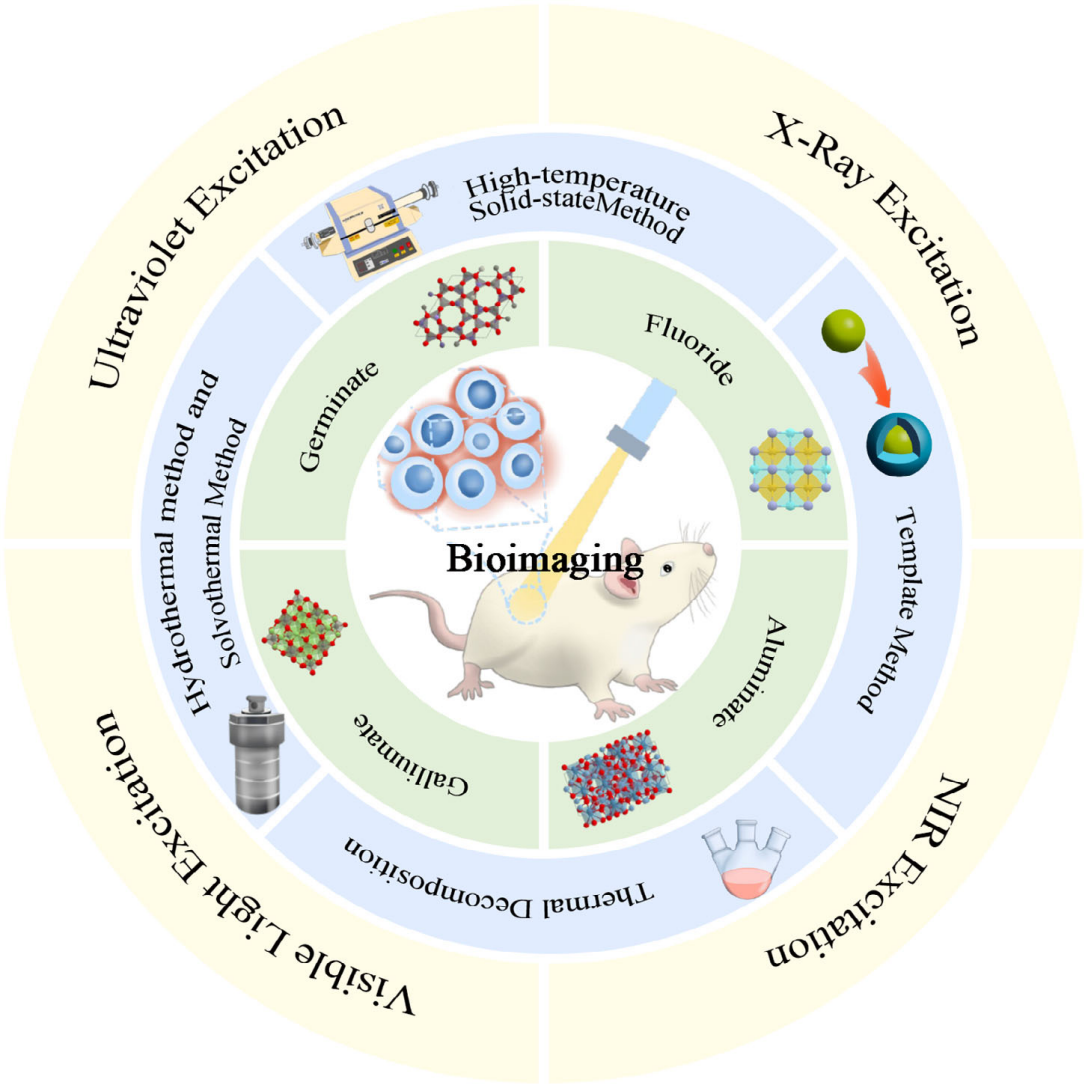
Synthesis, mechanism, and bioimaging of inorganic persistence phosphors.

The rapid development of bioimaging has continuously expanded the application of inorganic persistence phosphors.^[^ ^12^, ^13^, ^14^, ^15^ ^]^ Inorganic persistence phosphors exhibit promising applications in the bioimaging fields due to the deep penetrating light sources, negligible autofluorescence, desirable signal‐to‐noise ratio, and sensitivity.^[^ ^16^, ^17^, ^18^ ^]^ Especially, the NIR window has higher imaging accuracy and better therapeutic effect in deep tissues.^[^ ^19^, ^20^, ^21^, ^22^, ^23^, ^24^, ^25^ ^]^ Herein, we mainly focus on an advanced review regarding inorganic persistence phosphors including the synthesis methods, mechanism, and representative matrixes for cancer diagnosis. (Scheme 1). Importantly, we preliminarily expound on the biosafety issues of inorganic persistence phosphors. Finally, we discuss the prospects and challenges for constructing and fabricating new‐generation inorganic persistence phosphors for bioimaging.

## 2 SYNTHESIS METHOD

Bioimaging usually requires materials with suitable dimensions to ensure their specific optical, thermal, and magnetic properties in living organisms.^[^ ^26^, ^27^, ^28^, ^29^ ^]^ To make the inorganic persistent phosphors more compatible with the requirements, several representative preparation strategies have been extensively investigated and modified for transformation, such as high‐temperature solid state,^[^ ^30^, ^31^, ^32^ ^]^ thermal decomposition,^[^ ^33^, ^34^ ^]^ hydrothermal/solvothermal method,^[^ ^35^, ^36^ ^]^ and template method.^[^ ^37^ ^]^ Noticeably, the structure and composition, luminescence intensity and lifetime, emission wavelength, and half‐peak width of inorganic persistence phosphors could be precisely regulated with the assistance of the aforementioned methods.^[^ ^38^, ^39^, ^40^ ^]^ In addition, sol–gel,^[^ ^41^, ^42^, ^43^ ^]^ and microwave methods^[^ ^44^ ^]^ have been utilized to produce the inorganic persistence phosphors, there is still a long way to go to optimize the method to obtain small size phosphors suitable for bioimaging. Herein, we try to find the suitable method to prepare desired inorganic persistent phosphors, especially in the hope of obtaining inorganic persistent phosphors with appropriate size, good fluorescence properties, and satisfactory biosafety.^[^ ^45^, ^46^, ^47^ ^]^

### 2.1 High‐temperature solid state

**FIGURE 1:**
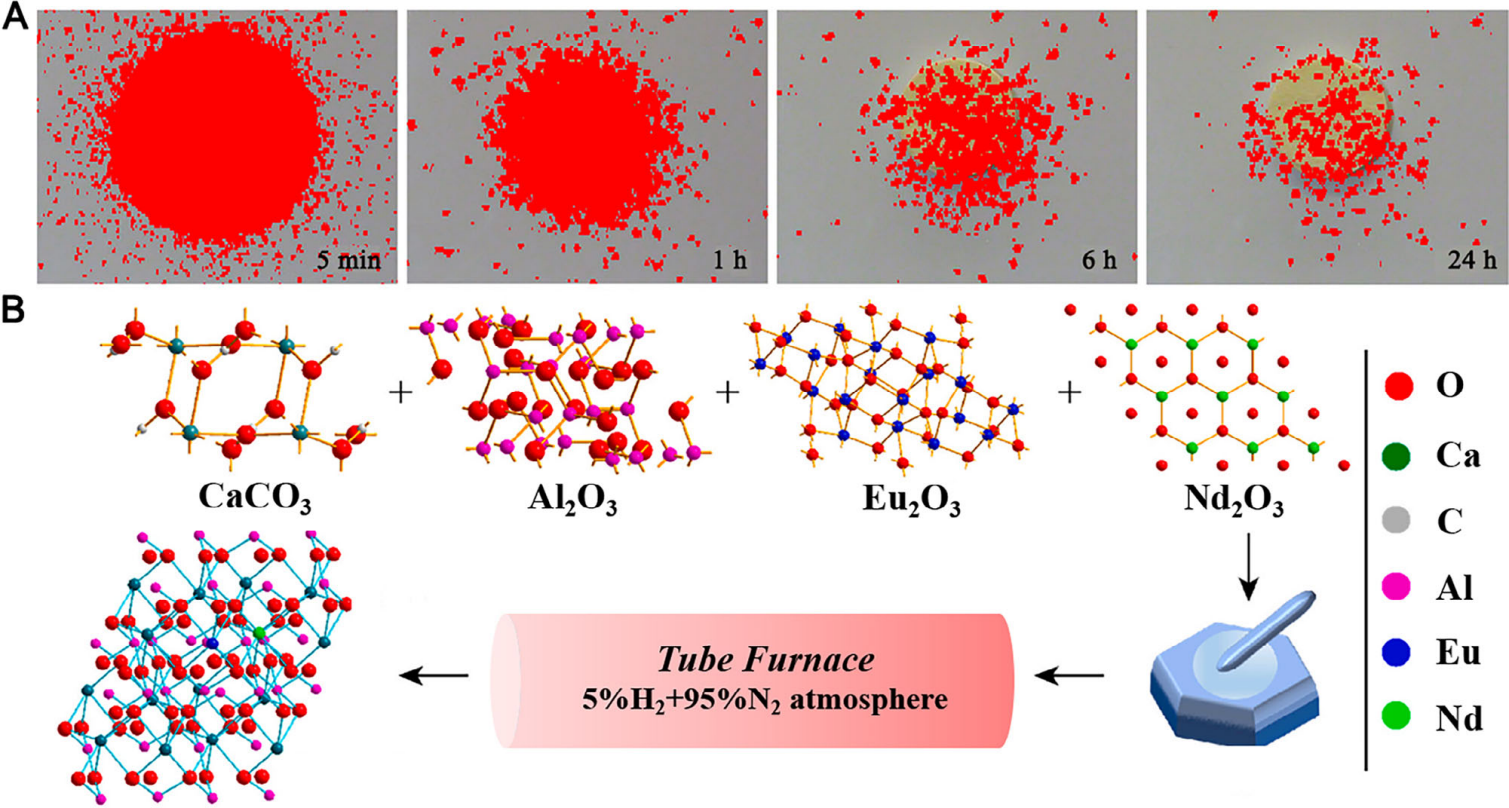
Synthesis of persistence phosphors by high‐temperature solid state method. (A) Ultraviolet‐C images of a decaying Ca_2_Al_2_SiO_7_:Pr^3+^ disc in room sunlight. Reproduced with permission.^[^ ^64^ ^]^ Copyright 2020, Springer Nature. (B) Morphology and structure characteristics of CaAl_2_O_4_:Eu,Nd. Reproduced with permission.^[^ ^71^ ^]^ Copyright 2021, Elsevier.

For the rapid proliferation of the traditional high‐temperature solid state method, it is easy to obtain inorganic persistence phosphors with the large size and high luminous efficiency.^[^ ^48^, ^49^, ^50^, ^51^ ^]^ The high temperature can effectively soar the intrinsic concentration of defects to prolong the afterglow time of inorganic persistence phosphors.^[^ ^52^, ^53^ ^]^ Some typical examples of high‐temperature solid‐state synthesis of inorganic engineering persistence phosphors with long the afterglow time have been added in the Table 1. For instance, Wang et al. synthesized melilite‐structured Ca_2_Al_2_SiO_7_:Pr^3+^ persistent phosphors for self‐sustained glowing tags by the high‐temperature solid state methods.^[^ ^64^ ^]^ Interestingly, Pr^3+^ ions are eightfold coordinated in the aforementioned persistent phosphors due to the substitution of Pr^3+^ ions for Ca^2+^ ions. The highly coordinated and charge‐imbalanced cation sites can offer a strong crystal field for Pr^3+^ ions. And cation size mismatch and charge imbalance are expected to create more oxygen vacancies around Pr^3+^ ions, which is required for the persistence phosphors. Therefore, Ca_2_Al_2_SiO_7_:Pr^3+^ persistent phosphors featured the strong ultraviolet‐C images after 24 h decay in room light (Figure 1A).

**TABLE 1:** Examples of high‐temperature solid‐state methods.

Examples	Temperature	Afterglow time	Reference
LaGaO_3_:Sb^3+^,Cr^3+^	1350°C	500 h	[54]
LiGaO_2_:Mn^2+^	1300°C	48 h	[55]
MgGeO_3_:Yb^3+^	1250°C	100 h	[48]
LiYGeO_4_:Bi^3+^	1250°C	300 h	[56]
Sr_3_Y_2_Ge_3_O_12_:Bi^3+^	1250°C	60 h	[57]
Sr_3_SiO_5_:Eu^2+^,Nb^5+^	1450°C	14 h	[58]
Ba_2_SiO_4_:Eu^2+^,Ho^3+^	1350°C	48 h	[59]
NaBaScSi_2_O_7_:Tb^3+^	1300°C	12 h	[60]
Na_2_CaGe_5_SiO_14_:Cr^3+^	1050°C	10 h	[61]
Zn_2_SnO_4_:Cr^3+^	1350°C	435 h	[62]
Mg_2_InSbO_6_:Cr^3+^	1300°C	24 h	[63]

Generally, large‐size inorganic persistence phosphors with non‐uniform morphology could be obtained by the high‐temperature solid method. To reduce the large size, the top‐down method is used to achieve appropriate morphology and small size to expand the bio‐application in nanomedicine, such as photothermal therapy^[^ ^65^, ^66^, ^67^ ^]^ and photodynamic therapy.^[^ ^68^, ^69^, ^70^ ^]^ For instance, Chang et al. designed CaAl_2_O_4_:Eu,Nd persistence phosphors as the light source for photodynamic therapy.^[^ ^71^ ^]^ As exhibited in Figure 1B, CaAl_2_O_4_:Eu,Nd persistence phosphor with block‐like morphology is synthesized by high‐temperature solid state method under the H_2_ atmosphere in the presence of the corresponding oxides and carbonates. Then, PEGylation CaAl_2_O_4_:Eu,Nd is obtained via the NaOH etched and MeO‐PEG‐COOH modified. This strategy of non‐external irradiation based on PEGylation CaAl_2_O_4_:Eu,Nd persistence phosphor solves the problem of external light source in photodynamic tumor therapy.

### 2.2 Thermal decomposition

Persistence phosphors nanoparticles could be prepared by the thermal decomposition method. It is a common method to prepare nanoparticles by heating organometallic salt precursors in a high boiling point organic solvent. Rapid nucleation and grow of nanoparticles can be achieved by rapidly injecting the reaction feedstock into a high temperature surfactant solvent, and then controlling the morphology by time and temperature.^[^ ^72^, ^73^, ^74^ ^]^ Lv et al. reported an ultra‐small ZnGa_2_O_4_:Cr persistent luminescence nanodot by thermal decomposition method based on metal acetylacetone and oleylamine solvent under the argon atmosphere.^[^ ^75^ ^]^ ZnGa_2_O_4_:Cr persistent luminescence nanodot have uniform size distribution (average size, 5 nm), which promote the autofluorescence‐free deep‐tissue bioimaging of persistent nanophosphors.

### 2.3 Hydrothermal and solvothermal method

**FIGURE 2:**
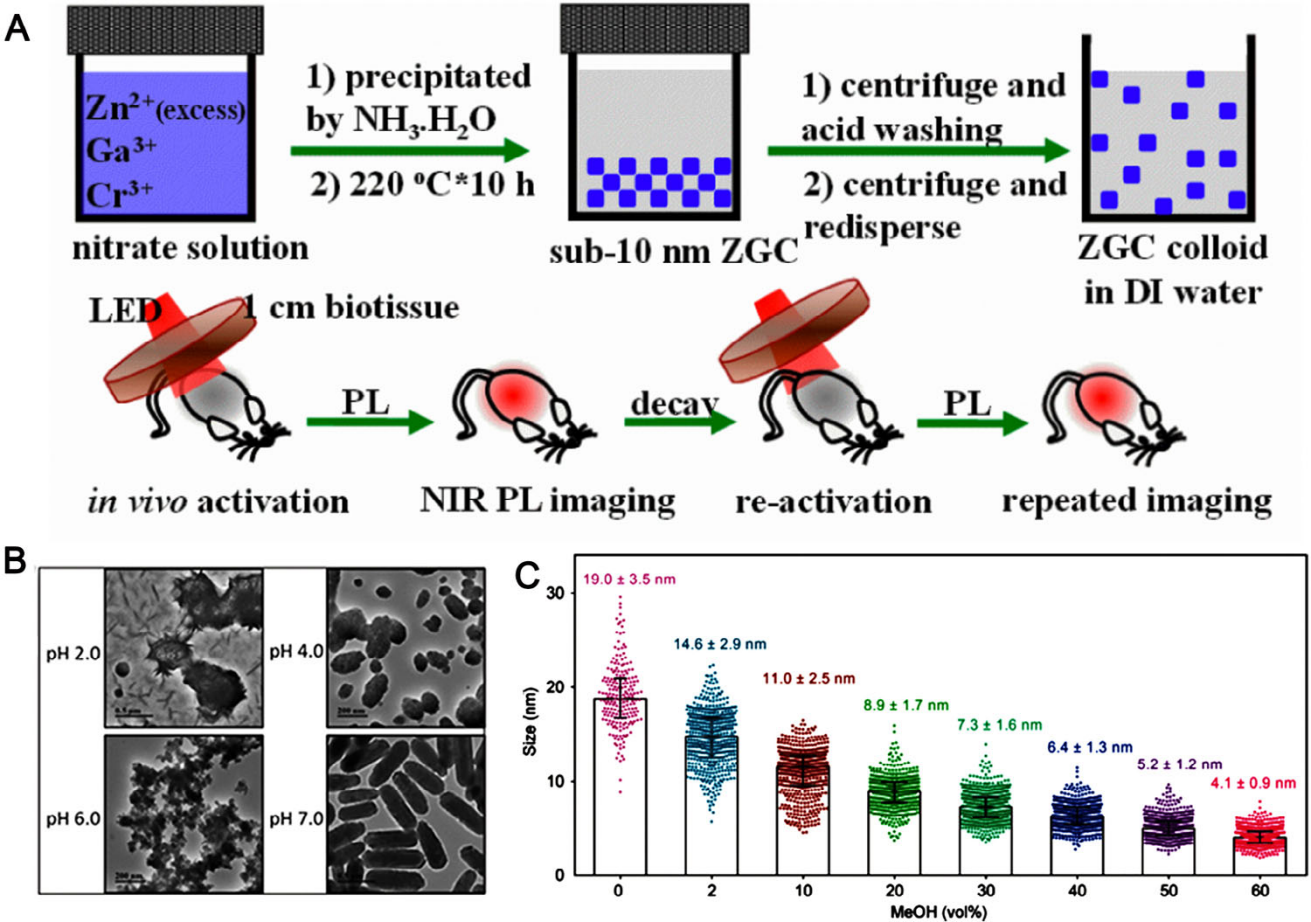
Hydrothermal and solvothermal synthesis of persistence phosphors. (A) Schematic illustration of the synthesis and imaging of ZnGa_2_O_4_:Cr^3+^ inorganic persistence phosphors. Reproduced with permission.^[^ ^86^ ^]^ Copyright 2015, American Chemical Society. (B) Synthesis and characterization of Zn_2_GeO_4_:Mn^2+^,Pr^3+^ inorganic persistence phosphors. Reproduced with permission.^[^ ^91^ ^]^ Copyright 2021, Wiley‐VCH. (C) Effect of CH_3_OH on the luminescence properties of Cr^3+^‐doped ZnGa_2_O_4_ nanoparticles. Reproduced with permission.^[^ ^95^ ^]^ Copyright 2020, American Chemical Society.

Varieties of persistence phosphors have been synthesized with high crystallinity, uniform size, and good persistent luminescent performance by the hydrothermal and solvothermal methods.^[^ ^76^, ^77^ ^]^ Some representative examples of hydrothermal and solvothermal methods of inorganic engineering persistence phosphors with nanoscale have been added in the Table 2. Hydrothermal method is one of the widely used nanocrystalline preparation techniques. For instance, Li et al. reported the monodisperse sub‐10 nm ZnGa_2_O_4_:Cr^3+^ near‐infrared persistent luminescence nanoparticles for deep tissue imaging (Figure 2A).^[^ ^86^ ^]^ In this system, the size of ZnGa_2_O_4_:Cr^3+^ could be adjusted by the composition of the precursor, especially the molar ratio of Zn and Ga. Interestingly, ZnGa_2_O_4_:Cr^3+^ nanoparticles possess intense persistent luminescence properties and good colloidal stability, which is a premise for biomedical applications. Despite progress in addressing the small size, the development of bioimaging using persistent phosphors has been hindered by their non‐degradability.^[^ ^87^, ^88^, ^89^, ^90^ ^]^ To surmount the obstacles, Li et al. reported the pH stimuli‐responsive luminescent behavior of Zn_2_GeO_4_:Mn^2+^,Pr^3+^ nanoparticles via a hydrothermal method.^[^ ^91^ ^]^ Interestingly, acid‐induced displacement reaction triggers Zn_2_GeO_4_:Mn^2+^,Pr^3+^ nanoparticles degradation (Figure 2B).

**TABLE 2:** Examples of hydrothermal and solvothermal methods.

Examples	Temperature	Size	Reference
ZnGa_2_O_4_:Cr^3+^	200°C	30–300 nm	[78]
SrAl_2_O_4_:Eu^2+^,Dy^3+^	170°C	100 nm	[79]
Cs_2_Na_x_Ag_1−_ *_x_* InCl_6_:Mn^2+^	180°C	2 nm	[80]
Y_2_O_2_S:Eu^3+^,M^2+^,Ti^4+^ (M = Mg, Ca, Sr, Ba)	180°C	80–150 nm	[81]
Zn_1.25_Ga_1.5_Ge_0.25_O_4_:Cr^3+^,Yb^3+^,Er^3+^	120°C	44.4 nm	[82]
Gd_2_O_2_S:Eu^3+^	200°C	100–200 nm	[83]
SnO_2_:Sm^3+^,Zr^4+^	180°C	500–700 nm	[84]
ZnGa_2_O_4_:Cr^3+^	220°C	32.7 nm	[85]

Except for hydrothermal method, the solvothermal method has been received extensive attention and in‐depth exploration research. In this method, the organic solvent, metal‐organic precursor, and reaction temperature play a decisive role in the morphology and size of the product.^[^ ^92^, ^93^, ^94^ ^]^ Wei et al. reported size‐tuned ZnGa_2_O_4_:Cr nanoparticles via solvothermal approach through methanol mediated.^[^ ^95^ ^]^ Interestingly, as the volume fraction of MeOH gradually increased from 0% to 60%, the size of the nanoparticles decreased from 19.0 ± 3.5 to 4.1 ± 0.9 nm (Figure 2C), because MeOH could decrease the solution viscosity and increase the solubility of the precursors. It may provide a strategy for obtaining ZnGa_2_O_4_:Cr nanoparticles with long afterglow under mild conditions.

### 2.4 Template method

**FIGURE 3:**
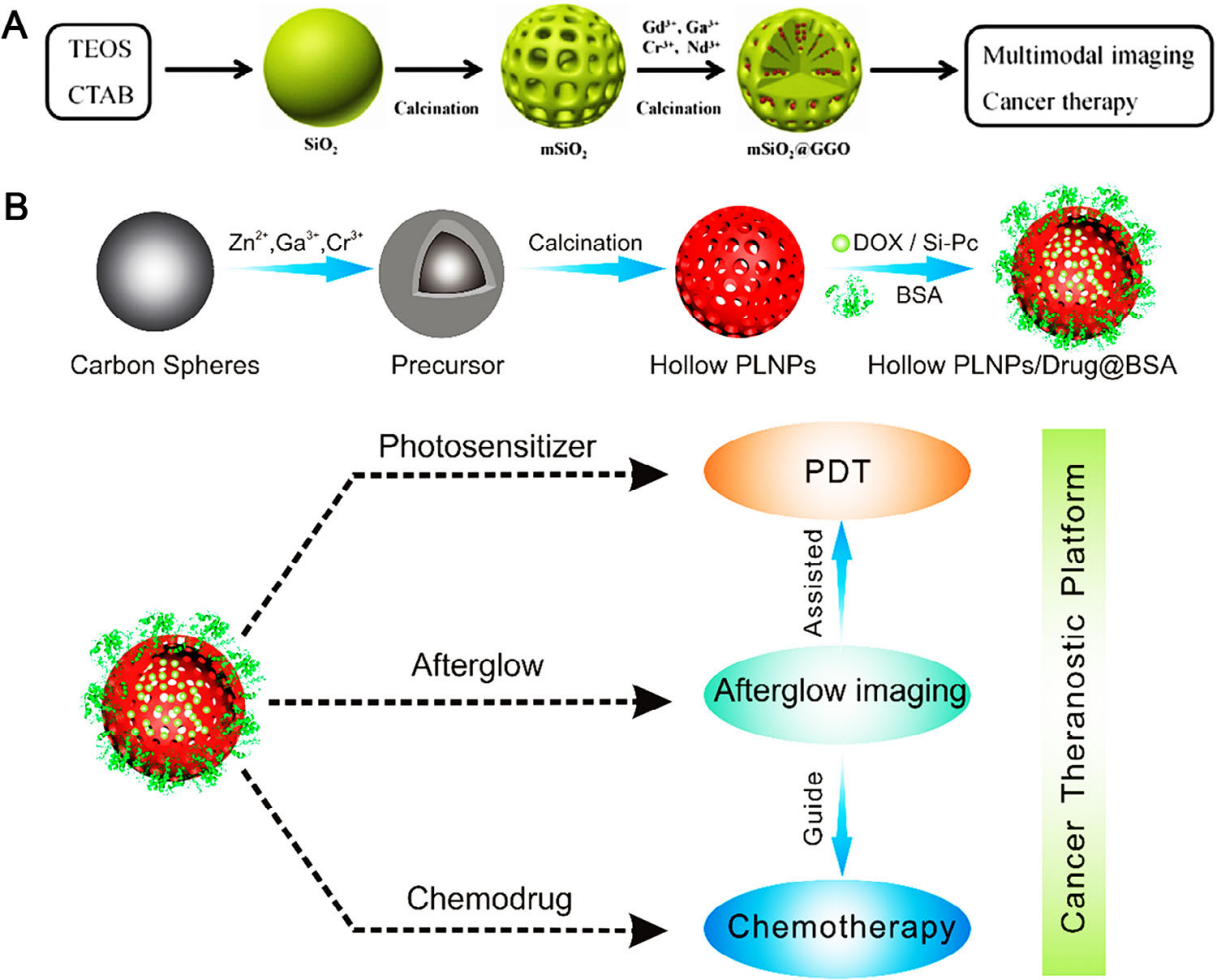
Synthesis of persistence phosphors by template method. (A) Schematic diagram of mSiO_2_@GGO persistence phosphors for multimodal imaging and cancer therapy. Reproduced with permission.^[^ ^96^ ^]^ Copyright 2018, Elsevier. (B) Synthesis, functionalization and application of hollow persistent luminous nanoparticles. Reproduced with permission.^[^ ^98^ ^]^ Copyright 2018, American Chemical Society.

Template method could accessibly obtain the controllable morphology and size of the product, such as the representative silica nanospheres template. Mesoporous Silica nanospheres possess some excellent features including favorable optical transparency, uncomplicated synthesis strategy, negligible cost, and repeatable coating/etching. For example, Shi et al. used mesoporous silica nanoparticles as template, and added the precursor solution to the multi‐channel of the template, and calcinated to obtain regular spherical mSiO_2_@GGO persistence phosphors for multimodal imaging and cancer therapy.^[^ ^96^ ^]^ In this system, as shown in Figure 3A, silica nanospheres are the framework for controlling the morphology. Cr^3+^ ions could act as emission centers for persistent luminescence at the first near‐infrared window, Nd^3+^ ions were used as emission centers in the second near‐infrared window. Except for mesoporous silica nanoparticles, carbon spheres template are also used to synthesize uniform nanoparticles.^[^ ^97^ ^]^ As exhibited in Figure 3B, Wang et al. creatively proposed large hollow cavity inorganic persistence phosphors based on carbon spheres template for tumor afterglow imaging and chemical/photodynamic therapies. This proof‐of‐concept study of hollow‐structured inorganic persistence phosphors could extend the applications in nanomedicine.^[^ ^98^ ^]^

## 3 MECHANISM

**FIGURE 4:**
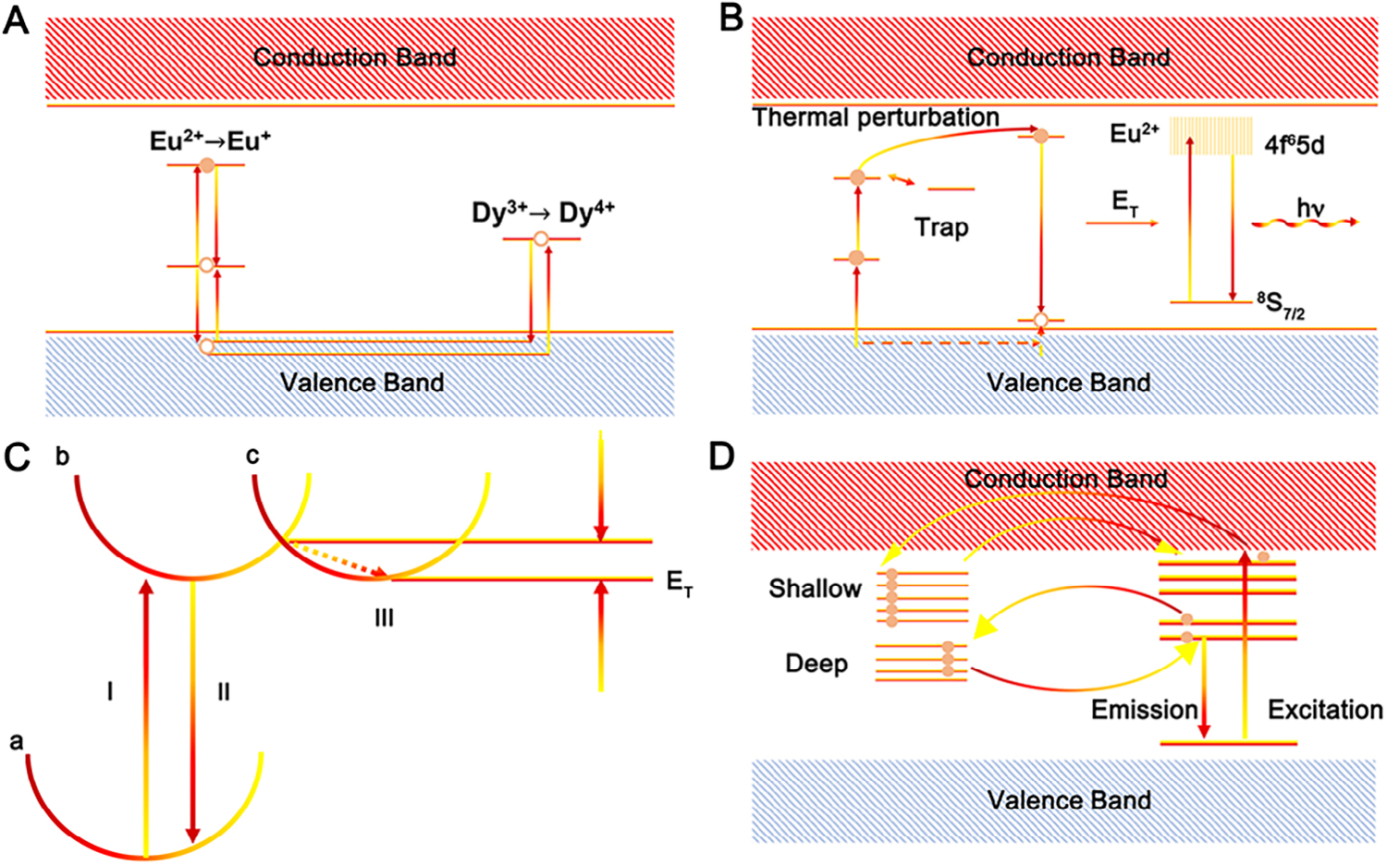
Persistent luminescence mechanism models. (A) The model of hole transport. Reproduced with permission.^[^ ^101^ ^]^ Copyright 1996, IOP Publishing. (B) The model of two‐photon oxygen vacancy. Reproduced with permission.^[^ ^103^ ^]^ Copyright 2001, Elsevier. (C) The model of displacement coordinate. (D) The model of tunneling effect. Reproduced with permission.^[^ ^105^ ^]^ Copyright 2015, American Chemical Society.

The persistence properties of persistence phosphors are determined by two factors: trap and luminescence center.^[^ ^99^, ^100^ ^]^ The trap center determines the afterglow time and luminescence intensity. Due to the complexity of the long afterglow luminescence process, researchers have proposed a variety of luminescence models for different matrixes, such as hole transport, two‐photon oxygen vacancy, displacement coordinate, and tunneling effect models. As displayed in Figure 4, the hole transport model assumes that the hole is the main charge carrier. When Eu^2+^ is excited by photons, a hole escapes to valence band, and the hole is captured by rare earth ions such as Dy^3+^, and the captured hole is released to the c by heat energy.^[^ ^101^, ^102^ ^]^ The two‐photon oxygen vacancy model captures electrons by using oxygen vacancies as electron traps, which is especially suitable for the long afterglow matrix of oxides.^[^ ^103^ ^]^ The displacement coordinate model is a widely accepted mechanism model.^[^ ^104^ ^]^ Under the excitation of the external light source, the electrons transit from the ground state to the excited state, and some electrons directly transit back to the ground state, resulting in the luminescence. The other part of the electrons are captured by the trap energy level. This part of the electrons absorb enough energy to overcome the energy gap between the trap and the excitation energy level, and will be released back to the ground state to produce the glowing phenomenon. The tunneling model refers to the ion passing through a barrier that is traditionally inaccessible. This model no longer requires a close energy level and better explains the deep trap‐related long afterglow matrix.^[^ ^105^, ^106^ ^]^

### 3.1 Ultraviolet

Ultraviolet (UV) light is the most common excitation source for persistence phosphors. Suitable structure and band gap play important roles in afterglow performance. Luo et al. proposed that the structure and defects of Mn^2+^‐doped calcium aluminum germanate photonic glasses could be controlled by the amorphous structure continuity and facile network topology tuning strategy.^[^ ^107^ ^]^ In addition, Miao et al. reported Sr_3_Sc_2_Ge_3_O_12_:Bi^3+^ exhibited the long‐lasting ultraviolet‐A (UVA) persistent luminescence after UV excitation at 254 nm.^[^ ^108^ ^]^ The ground state electrons of the luminescent ions are excited to the conduction band under the excitation of 254 nm ultraviolet light, and some of the excited electrons are trapped by the energy trap. As the irradiation time increases, the energy traps are completely filled and the deep traps are filled by the nonradiative relaxation of the shallow traps. After stopping excitation, the stored electrons escape back to the emission state. Radiation recombination with the capture hole at Bi^3+^ leads to UVA persistent luminescence.

### 3.2 Visible light

**FIGURE 5:**
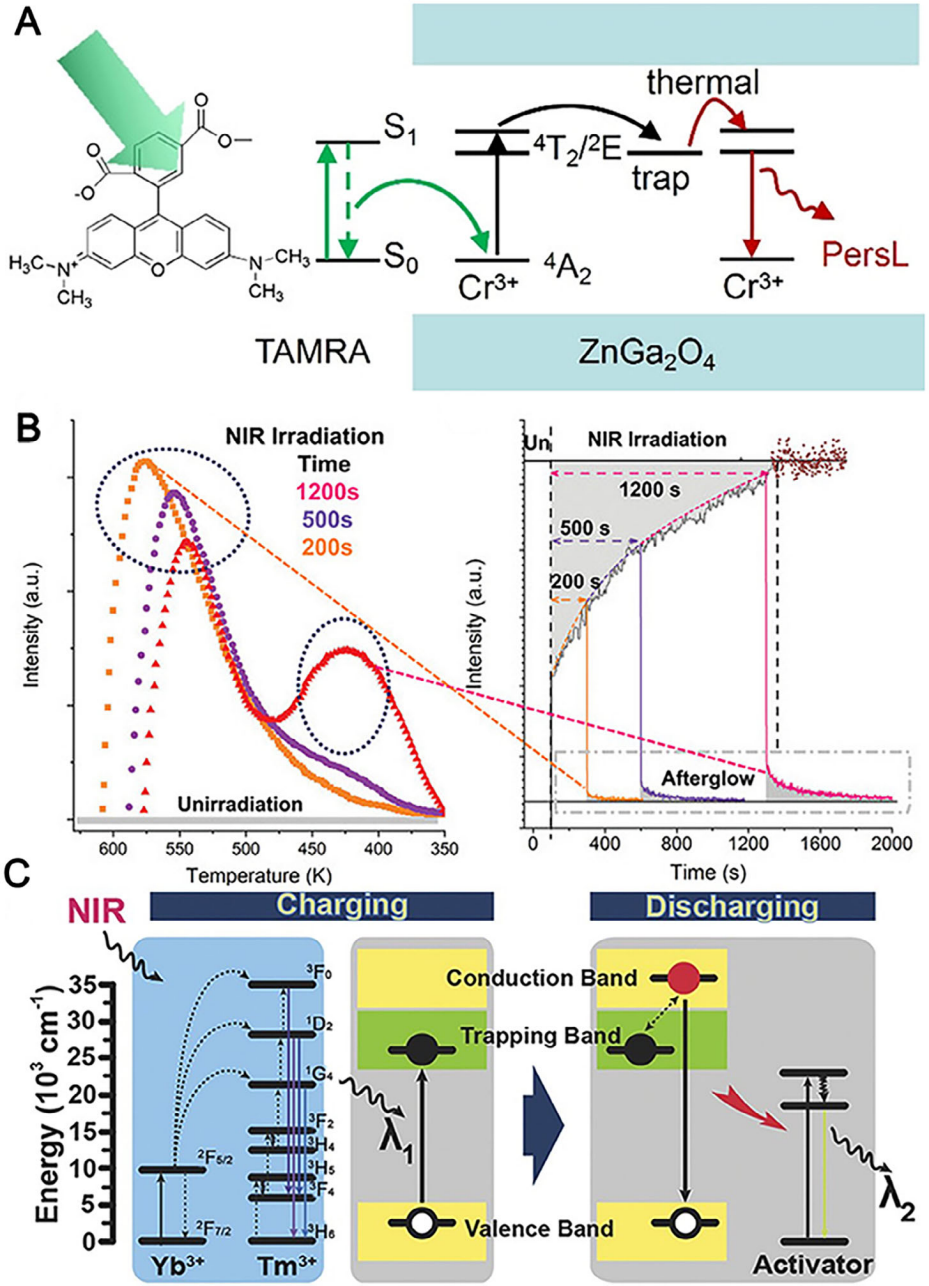
The mechanism of persistent luminescence upon different excitation. (A) The mechanism on the enhanced PersL of Tamra‐sensitized persistent luminescence nanoparticle. Reproduced with permission.^[^ ^109^ ^]^ Copyright 2021, Wiley‐VCH. (B) Thermoluminescence spectra after NIR irradiation and kinetic processes during NIR irradiation. Reproduced with permission.^[^ ^121^ ^]^ Copyright 2021, Wiley‐VCH. (C) The energy‐transfer mechanism of upconverting persistent luminescence. Reproduced with permission.^[^ ^122^ ^]^ Copyright 2017, Wiley‐VCH.

Visible light activatable ZnGa_2_O_4_:Cr^3+^ persistent luminescence nanoparticle has been emerging for bioimaing. For instance, Li et al. select to combine functionalized ZnGa_2_O_4_:Cr^3+^ with organic Rhodamine dye (TAMRA) to create dye‐sensitized persistent phosphors.^[^ ^109^ ^]^ TAMRA has the intense absorption within the long‐wavelength recharging window of ZGC persistent luminescence nanoparticle. And the fluorescence emission of TAMRA overlaps well with the ^4^A_2_ to ^4^T_2_ of Cr^3+^ ions in the ZnGa_2_O_4_. Then, TAMRA could absorb the 560 nm light and transfer the energy to Cr^3+^ions, resulting in the long afterglow luminescence (Figure 5A).

### 3.3 Near infrared light

Although the long afterglow material can obtain good luminous effect under ultraviolet light or visible light or X‐ray excitation, the ultraviolet light or visible light suffer from suboptimal tissue absorption and the use of X‐ray exposure is inconvenient. In contrast, near infrared light (NIR) feature the improved deep tissue permeation and reduced optical damage and toxicity, showing strong application potential in bioimaging.^[^ ^110^, ^111^, ^112^, ^113^, ^114^, ^115^, ^116^, ^117^, ^118^, ^119^, ^120^ ^]^ Chen et al. prepared a NIR rechargeable persistence phosphors due to the better biological applicability of NIR photons. CaSnO_3_:Bi^3+^ can create “upconversion‐like” trap energy storage at continuously distributed defect points.^[^ ^121^ ^]^ According to theoretical simulation results (Figure 5B), there are two distinct trap bands with varying depths that enable defect trap energy storage and upconversion‐mediated near‐infrared luminescence. These findings offer potential new avenues for the biological design of persistent phosphors. Similarly, Hu et al. explored the luminescence mechanism of NIR rechargeable upconversion inorganic persistence phosphors.^[^ ^122^ ^]^ During the charging process, the ultraviolet/blue upconversion emission photons of NaYF_4_:Yb,Tm are absorbed by the persistence phosphors matrix and filled into the excited state level, resulting in energy storage in the electron trap. Upon the 980 nm excitation, the energy captured by the trap is released and transferred to the activator, thereby achieving long afterglow luminescence (Figure 5C).

### 3.4 X‐ray

X‐ray excited persistent phosphors have attracted extensive attention and research. For instance, Huang et al. observed that the Zn_2_GeO_4_:Mn persistent phosphors has better X‐ray excitation ability when Li^+^ replaces Zn^2+^.^[^ ^123^ ^]^ Due to the simple electronic structure of Li^+^, it is more prone to effective photoionization. The absorbed high‐energy X‐ray photons can emit high‐energy electrons, further producing secondary high‐energy electrons. These electrons can be excited in the conduction band to charge the electron traps. Meanwhile, Xue et al. proposed an X‐ray activated ZnGa_2_O_4_:Cr with efficient near‐infrared long afterglow luminescence.^[^ ^124^ ^]^ The X‐ray excitation energy is absorbed by the ZnGa_2_O_4_ and Cr^3+^, which leads to the electron transition ^4^A_2_ → ^4^T_1_ of Cr^3+^ and produces the NIR persistent luminescence.

## 4 REPRESENTATIVE MATRIX

### 4.1 Galliumate

Galliumate systems have been attracted great attention in the application of NIR persistent luminescence imaging with help of the Cr^3+^ ions emission centers.^[^ ^125^ ^]^ In ZnGa_2_O_4_, Cr^3+^ ions can replace Ga^3+^ ions in slightly triangularly distorted octahedral sites, constituting afterglow occurs by excitating Cr^3+^ ions. For instance, the mSiO_2_@Zn_1.05_Ga_1.9_O_4_:Cr@HKUST‐1 (HSZGO) has been used for bioimaging and tumor synergic therapy.^[^ ^126^ ^]^ In this system, the NIR PersL signals of HSZGO could be monitored after 30 min, which is more suitable for bioimaging.

**FIGURE 6:**
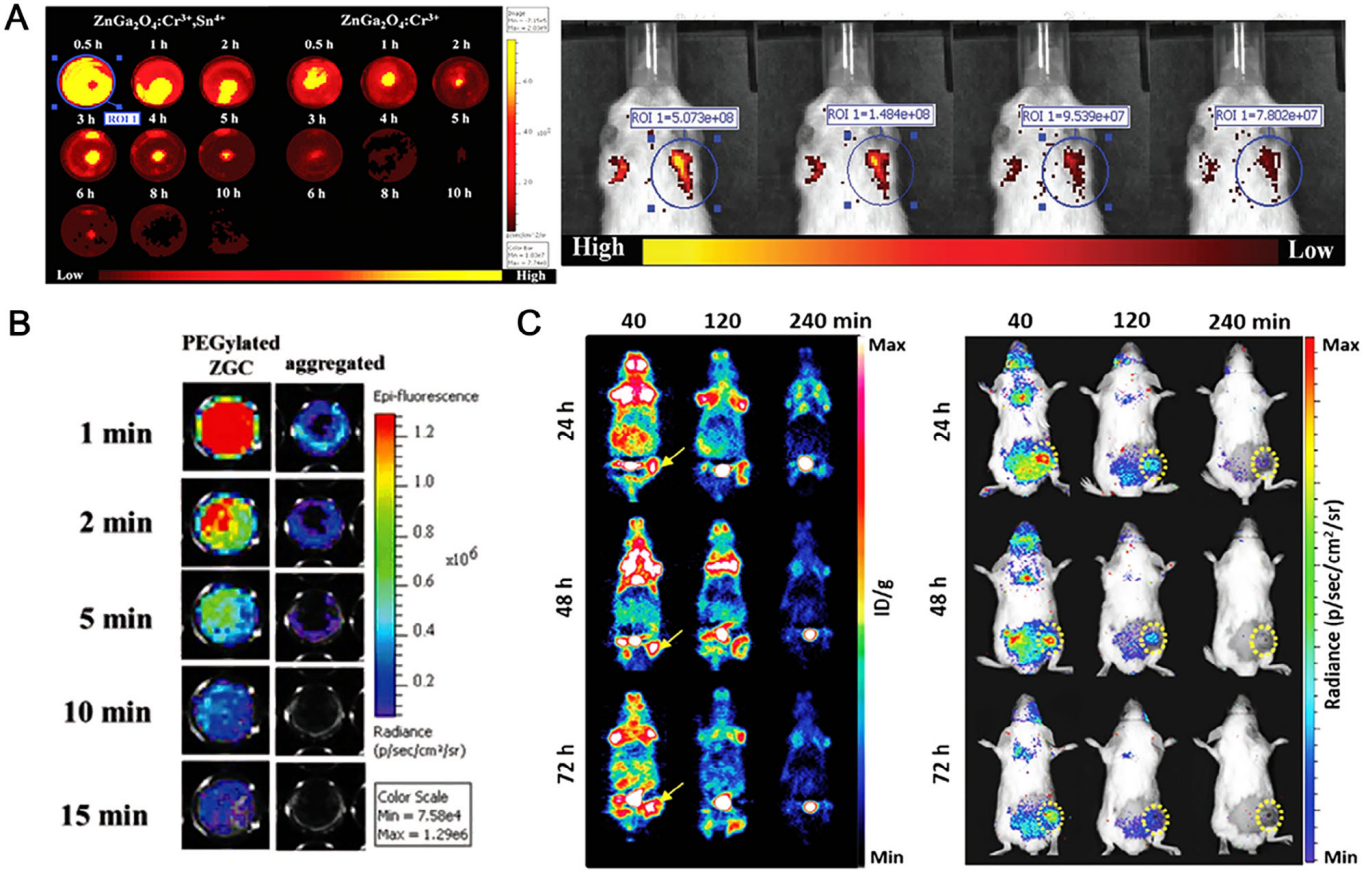
Application of galliumate persistent phosphor. (A) The persistent luminescence of ZnGa_2_O_4_:Cr^3+^,Sn^4+^ and the near infrared‐persistent fluorescent bioimaging signal of AFT‐PLN@MAp. Reproduced with permission.^[^ ^127^ ^]^ Copyright 2020, Wiley‐VCH. (B) The long‐lasting luminescence images of PEGylated ZGC nanocubes and aggregated nanoparticles after X‐ray excitation ceased. Reproduced with permission.^[^ ^128^ ^]^ Copyright 2019, Wiley‐VCH. (C) Representative wholebody coronal PET images and luminescence images of 4T1 tumor‐bearing mice after administration of ^18^F‐FDG. Reproduced with permission.^[^ ^129^ ^]^ Copyright 2020, Wiley‐VCH.

It is noteworthy that the addition of Sn^4+^ ions can enhance the NIR afterglow luminescence of Cr^3+^ ions in ZnGa_2_O_4_ by providing additional electron traps. For instance, AFT‐PLN@MAp lung cancer therapeutic nanoplatforms are constructed by ZnGa_2_O_4_:Cr^3+^,Sn^4+^, mSiO_2_, afatinib target drugs, and MAGE‐A3 targeting inducer.^[^ ^127^ ^]^ As shown in Figure 6A, ZnGa_2_O_4_:Cr^3+^,Sn^4+^ exhibits the better NIR persistent luminescence than ZnGa_2_O_4_:Cr^3+^, because the adjacent energy levels of Sn^4+^ and Cr^3+^ ions can form electron traps, keeping high‐energy electrons in excited states for longer periods of time, the excess energy storage space could also improve the luminous efficiency of NIR afterglow luminescence. Importantly,

The NIR persistent luminescence signal was repeatedly observed in the lung at least 6 h (Figure 6A), which may provide an alternative strategy for lung cancer diagnosis and treatment.

Unlike light as an excitation source, the X‐ray can also induce intense long‐lasting luminescence. Chen et al. synthesized the X‐ray‐excited PEGylated ZnGa_2_O_4_:Cr^3+^ (ZGC) long afterglow luminescence concave nanocubes.^[^ ^128^ ^]^ Compared with the agglomerative ZnGa_2_O_4_:Cr^3+^, PEGylated ZGC feature much greater emission intensity and longer‐lasting luminescence after excitation ceased at 0.5 Gy (Figure 6B). In addition, ^18^F‐fluorodeoxyglucose (^18^F‐FDG) radiopharmaceutical can also be used to excite the persistent luminescence of ZnGa_2_O_4_:Cr^3+^. Figure 6C shows that ZnGa_2_O_4_:Cr^3+^ exhibits obvious longer‐lasting luminescence under the irradiation of ^18^F‐FDG, probably due to the Cerenkov resonance energy transfer, additional *γ*‐scintillation property, and persistent luminescence properties.^[^ ^129^ ^]^

### 4.2 Germanate

**FIGURE 7:**
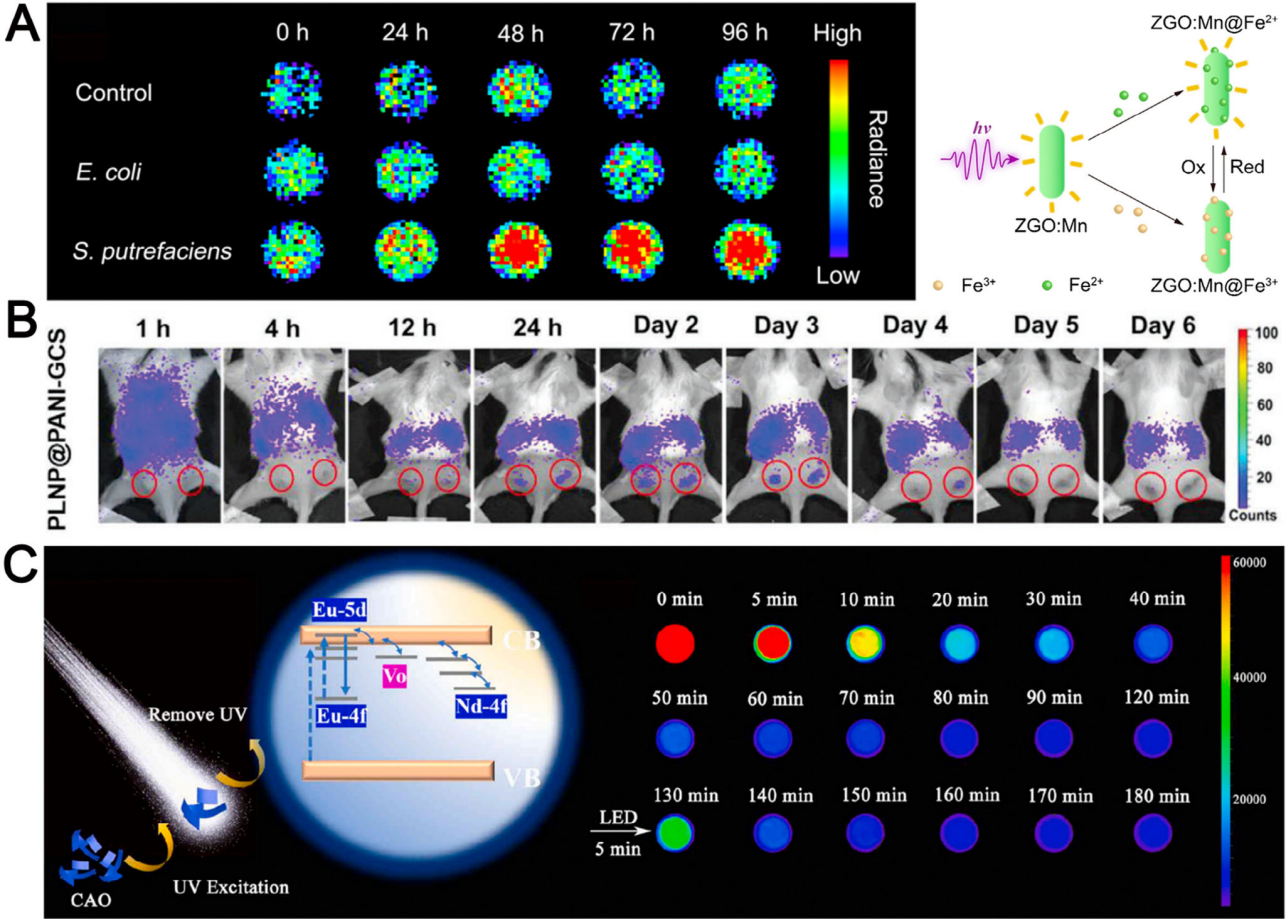
Application of germanate and aluminate persistent phosphor. (A) Persistent luminescence decay images of ZGO:Mn@Fe^3+^ upon exposure to *Shewanella putrefaciens* and *Escherichia coli*. Reproduced with permission.^[^ ^133^ ^]^ Copyright 2022, Wiley‐VCH. (B) Time‐dependent in vivo persistent luminescence images after intravenous injection of PLNP@PANI‐GCS. Reproduced with permission.^[^ ^143^ ^]^ Copyright 2020, Wiley‐VCH. (C) Schematic diagram of the persistent luminescence mechanism of CaAl_2_O_4_:Eu,Nd and the persistent luminescence images at different time intervals. Reproduced with permission.^[^ ^144^ ^]^ Copyright 2022, Elsevier.

Germanate system is also used as the matrix material for persistence phosphors.^[^ ^130^, ^131^, ^132^ ^]^ Zn_2_GeO_4_ has the typical phenacite structure, which can serve as the potential host material. Recently, Chen et al. reported an electron‐induced glow probe Zn_2_GeO_4_:Mn@Fe^3+^ (ZGO:Mn@Fe^3+^) for monitoring Fe (III) respiratory metabolism.^[^ ^133^ ^]^ Compared with Fe^2+^ ions, the persistent luminescence of Zn_2_GeO_4_:Mn is quenched by Fe^3+^. Interestingly, the quenched persistent luminescence of Zn_2_GeO_4_:Mn@Fe^3+^ could be recovered when Fe^3+^ accepted electrons from the dynamic Fe(III) respiration metabolism. Then, ZGO:Mn@Fe^3+^ is incubated with *Shewanella putrefaciens* that involves Fe(III) respiration metabolism and *Escherichia coli* without Fe(III) respiration metabolism. As displayed in Figure 7A, the persistent luminescence intensity of the ZGO:Mn@Fe^3+^ in *S. putrefaciens* group increased with incubation time, indicating that the specific for Fe(III) respiration metabolism monitoring. As a result, the key indicators of cytoplasmic electron transfer to extracellular receptors during Fe(III) respiratory metabolism could be verified by ZGO: Mn@Fe^3+^.^[^ ^134^, ^135^, ^136^, ^137^ ^]^

Zinc gallogermanate has been employed for long glow luminescence. Zn_3_Ga_2_Ge_2_O_10_:0.5%Cr^3+^ with the glow life of more than 360 h.^[^ ^138^ ^]^ Yang et al. used Cr^3+^‐doped zinc gallogermanate superlong afterglow nanoparticles (ZGGO:Cr^3+^) for deep tissue temperature sensing. ZGGO:Cr^3+^ ratiometric luminescent nanothermometers feature the viable conversion potential for temperature sensing, even the tissue depth reaches 15 mm.^[^ ^139^ ^]^ In addition, NIR persistent materials have potential applications in imaging‐guided therapy.^[^ ^140^ ^]^ Photothermal therapy (PTT) depends on the high photothermal conversion efficiency of the photothermal agent. However, the uncontrollable distribution of photothermal agents limits the clinical transformation of PTT.^[^ ^141^, ^142^ ^]^ To overcome the challenge, Yan et al. developed a polyaniline (PANI) and glycol chitosan (GCS) functionalized Zn_1.2_Ga_1.6_Ge_0.2_O_4_:Cr^3+^@PANI‐GCS (PLNP@PANI‐GCS) for persistent luminescent bacterial infection imaging and precise photothermal therapy.^[^ ^143^ ^]^ In this system, Zn_1.2_Ga_1.6_Ge_0.2_O_4_:Cr^3+^ acts as the core for renewable NIR‐persistent luminescence, while PANI is employed as the shell for pH‐dependent photothermal agents. GCS acts as the water‐soluble biopolymer with pH‐dependent charge, improving the biocompatibility of PLNP@PANI‐GCS. PLNP@PANI‐GCS exhibits poor affinity to neighboring normal cells and has a limited photothermal effect in the normal physiological environment. As exhibited in Figure 7B, the Luminescence in the abscess area became significant at 24 h to 3 days after PLNP@PANI‐GCS injection. The long‐term imaging of PLNP@PANI‐GCS in the abscess sites provide valuable spatial information for PTT.

### 4.3 Aluminate

Aluminate systems are characterized by high afterglow intensity and stable chemistry. Chang et al. developed a 2D CaAl_2_O_4_:Eu,Nd‐verteporfin‐triphenylphosphine persistent luminescence nanoplatform for photodynamic tumor nanotherapy.^[^ ^144^ ^]^ The long blue afterglow emission time of the Eu^2+^ ions was enhanced with the assistance of Nd^3+^ ions. CaAl_2_O_4_:Eu,Nd feature with exceptional persistent luminescence performance (Figure 7C). Then, CaAl_2_O_4_:Eu,Nd acts as optical battery to induce photosensitizer verteporfin to generate singlet oxygen. Except for CaAl_2_O_4_, SrAl_12_O_19_:Cr^3+^,Ti^4+^ have also been used for deep tissue imaging.^[^ ^145^ ^]^ Compared with the SrAl_2_O_4_:0.5%Cr^3+^, SrAl_12_O_19_:0.5%Cr^3+^,0.5%Ti^4+^ exhibited the strong afterglow emission. Upon subcutaneous injection of SrAl_12_O_19_:0.5%Cr^3+^,0.5%Ti^4+^, the bright and long‐lasting afterglow luminescence signal was observed. However, due to the poor hydrophilicity and large size, the aluminate system faces obstacles in terms of its transformation and practical application.

### 4.4 Fluoride

**FIGURE 8:**
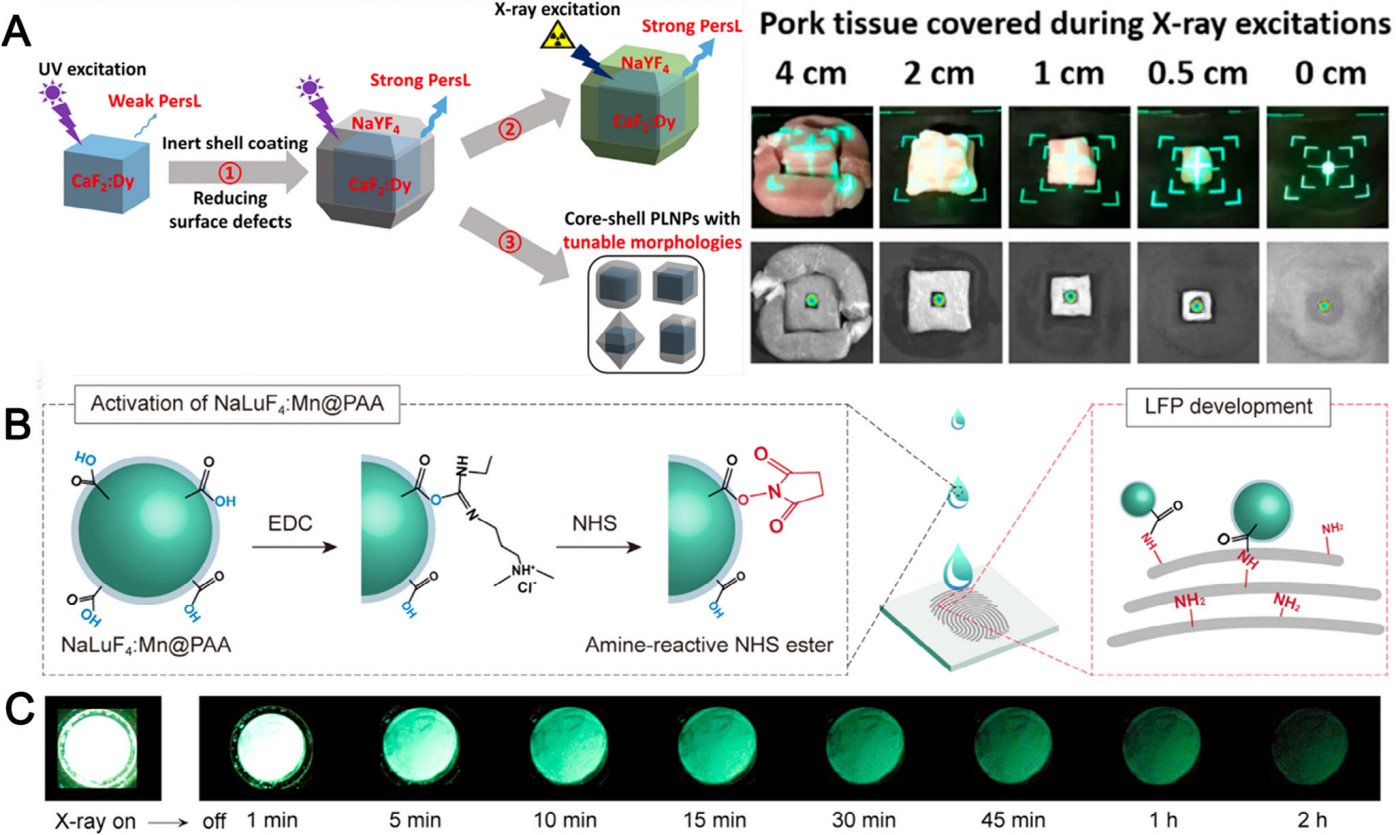
Application of fluoride persistent phosphor. (A) 3D controllable synthesis of CaF_2_:Dy@NaYF_4_, And the images and long afterglow images of CaF_2_:Dy@NaYF_4_ after X‐ray excitation of pork tissues with different thicknesses. Reproduced with permission.^[^ ^148^ ^]^ Copyright 2021, American Chemical Society. (B) Nanoparticles achieve LFP luminescence imaging by activation, and photographic images. Reproduced with permission.^[^ ^149^ ^]^ Copyright 2022, American Chemical Society. (C) Radioluminescence and long afterglow luminescence images of NaLuF_4_:Mn nanoparticles. Reproduced with permission.^[^ ^149^ ^]^ Copyright 2022, American Chemical Society.

Although galliumate or germanate systems have been attracted great attention in the application of NIR‐I imaging or visible light imaging, lanthanide‐ions‐doped fluorides have been studied as important luminescent nanomaterials in bioimaging, especially the NIR‐II imaging. In addition, aluminate system suffers from the poor hydrophilicity and large size. Therefore, among the representative paradigms, fluoride is more advantageous for cancer diagnostics.^[^ ^146^, ^147^ ^]^ Huang et al. prepared a CaF_2_:Dy@NaYF_4_ persistence phosphors, where Yb^3+^ and Er^3+^/Tm^3+^ lanthanide ions were doped to achieve both long afterglow and upconversion functions. This design was proven to be effective for X‐ray excitation of deep tissues, as demonstrated in Figure 8A.^[^ ^148^ ^]^ The engineering of inorganic persistent phosphors with tunable luminescence from X‐ray activated NIR‐II windows presents several challenges. Pei et al. synthesized persistent phosphors with core–shell structure, featuring dynamic information transfer. Moreover, the phosphors display minimal cytotoxicity and stability in biological media, which makes them promising candidates for long‐term nanoprobe tracking and monitoring. Likewise, the material demonstrates excellent long afterglow performance when lanthanum is replaced with lutetium. NaLuF_4_:Mn has a high‐precision latent fingerprint (LFP) of 1–3 levels (Figure 8B).^[^ ^149^ ^]^ The surface modification of polyacrylic acid endows the material with excellent dispersibility and enables it to exhibit strong afterglow luminescence upon X‐ray excitation, making it a promising candidate for bioimaging.

## 5 SUMMARY AND OUTLOOK

**FIGURE 9:**
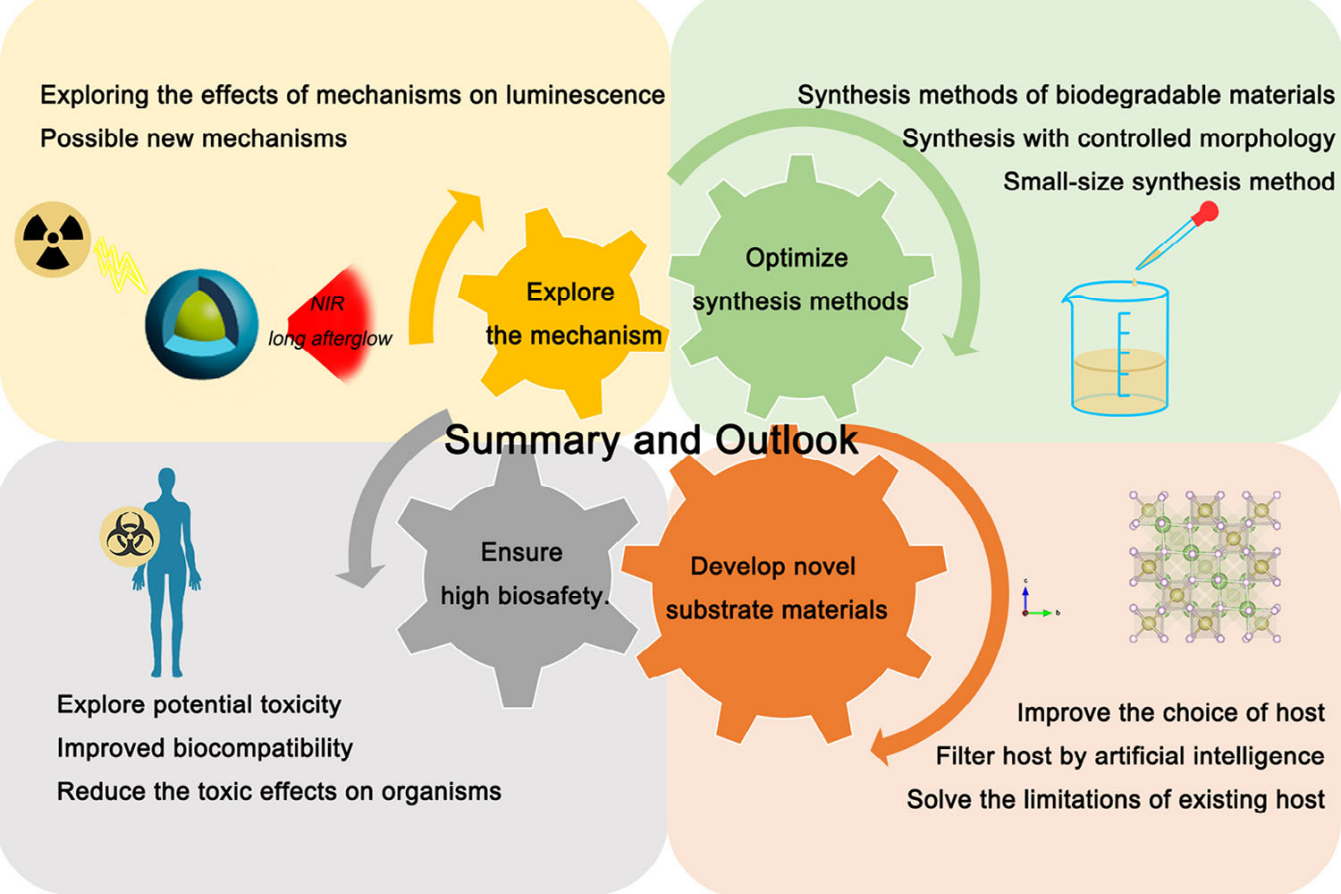
A summary program of persistence phosphors in cancer diagnostics.

In summary, persistence phosphors have achieved great advances, the main research goal is to prepare nano‐engineering inorganic persistence phosphors with excellent chemical stability, high luminescence intensity and long afterglow lifetime, biocompatibility.^[^ ^150^, ^151^, ^152^ ^]^ As shown in Figure 9, there are still several challenges that need to be addressed in order to facilitate their practical applications.

### 5.1 Optimize synthesis methods

To expand the potential applications of persistent phosphors, it is crucial to develop synthetic methods for producing biodegradable nanomaterials that are suitable for bioimaging. We have presented a comprehensive overview of several methods with promising potential. The purpose is to further refine the synthesis method based on these findings, which is critical for advancing the application of persistent phosphors.

### 5.2 Explore the mechanism

Although several typical models of the luminescence mechanism have been proposed, the luminescence mechanism of persistence phosphors is not fully understood at this stage. For example, it is not yet clear how the energy stored in the trap is conducted to the luminescence center, whether the charge carriers come from the luminescence center, the co‐dopant near the activator, and how lattice defects influence the luminescence behavior.

### 5.3 Develop novel substrate materials

Most research on inorganic persistence phosphors is focused on major materials such as galliumate, germanate, aluminate, and fluoride, which still have limitations in bioimaging. To address this, the substrate material selection can be improved by further exploring the composition, structure, valence state, and charge distribution of the materials. Additionally, artificial intelligence can be utilized to identify promising substrate materials. Exploring more abundant inorganic persistence phosphors matrix may be more suitable for biological organisms.

### 5.4 Ensure high biosafety

To enable clinical applications, it is necessary to consider potential toxic effects. Inorganic persistent phosphors can be synthesized to minimize toxic and side effects on organisms. Moreover, further studies are needed to investigate the mechanism and biodistribution of persistent phosphors.

Nano inorganic persistence phosphors have the potential to overcome autofluorescence and improve the signal‐to‐noise ratio and sensitivity in bioimaging. Our review presents new ideas and suggestions for the synthesis of the next generation of engineering inorganic persistence phosphors, which can promote and expand the application of bioimaging.

## CONFLICT OF INTEREST STATEMENT

The authors declare no conflicts of interest.
